# Nerve merging repair in the replantation of a severed limb with defects in multiple nerves: five cases and long-term follow-up

**DOI:** 10.1186/s12893-022-01673-1

**Published:** 2022-06-09

**Authors:** Wenquan Ding, Xueyuan Li, Hong Chen, Xiaofeng Wang, Danya Zhou, Xin Wang

**Affiliations:** Department of Hand Surgery, Department of Plastic Reconstructive Surgery, Ningbo No. 6 Hospital, 315040 Ningbo, China

**Keywords:** Magnified nerve regeneration, Nerve repair, Avulsion amputation, Autogenous nerve graft

## Abstract

**Background:**

Repairing all nerves is challenging in cases of upper arm avulsion combined with defects in multiple nerves because the donor area for autogenous nerve transplantation is limited and the outcomes of long-segment allogeneic nerve transplantation are poor. Based on the principle of magnified nerve regeneration, we present a method called nerve merging repair, the feasibility of which needs to be confirmed in clinical practice.

**Methods:**

The nerve merging repair method relies on the use of fewer proximal nerves to innervate more distal nerves and depends mainly on whether the radial nerve (RN) can repair itself. In the case of defects in multiple nerves precluding RN self-repair, median-(median + radial) (M-(M + R)) repair is performed. If the RN can undergo self-repair, median-(median + ulnar) (M-(M + U)) or ulnar-(ulnar + median) (U-(U + M)) is used to repair the three nerves. Five cases were included in the study and involved the analysis of joint motor function, muscle strength and sensory recovery of the affected limb.

**Results:**

The replanted limb survived in all 5 cases. Follow-up visits were conducted with the patients for 51–80 months, during which they experienced satisfactory recovery of skin sensation, elbow flexion and extension and partial recovery of hand muscle strength.

**Conclusions:**

To a certain extent, treatment with the nerve merging repair method improved the sensory and motor function of the affected limb and limited the loss of function of the donor nerve area. This intervention provides a new approach for repairing long-segment defects in multiple nerves caused by avulsion amputation of the upper limb.

## Background

Despite the posttreatment sequelae affecting the upper limb and the difficulty of regaining the preinjury level of function [1], patients’ level of satisfaction with replantation is still higher than that with wearing a prosthetic limb [2]. Replantation of a severed upper limb is a challenging medical problem in orthopaedic and hand surgery [3–5]. Avulsion amputation at the elbow or upper arm is accompanied by multiple nerve avulsions and defects [6]. Short-segment defects of blood vessels and nerves in the severed limb can be repaired by a shortening osteotomy [7]. However, in cases of multiple long-segment nerve defects, there may be inadequate autogenous donor nerves for grafting [8], and outcomes of large-segment allogeneic nerve transplantation are poor [9, 10]. Hence, in such cases, it is not feasible to perfectly repair each nerve of the severed limb [11].

**Fig. 1:**
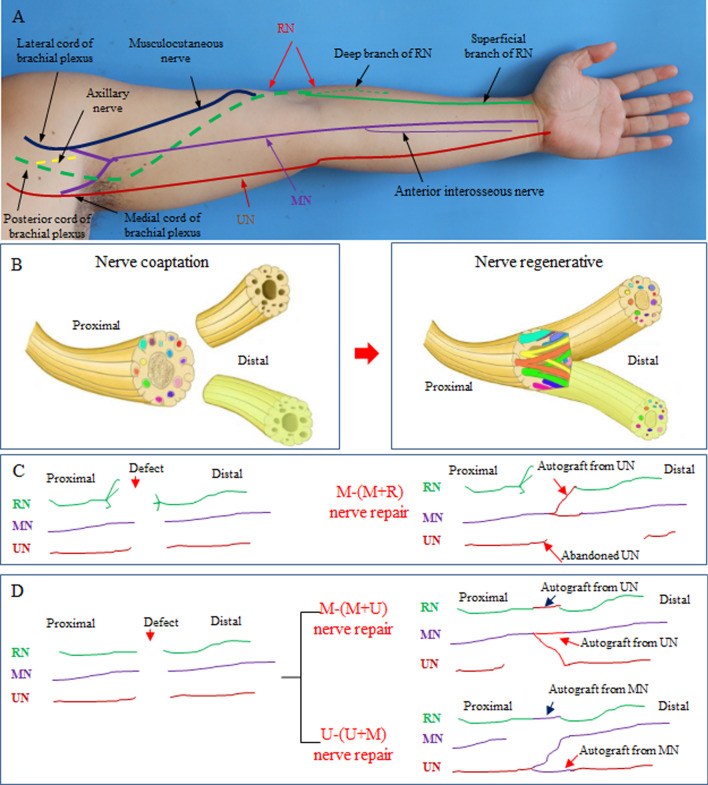
Illustrations of nerve merging repair. **A** Main nerves of the upper limb. **B** Magnified nerve regeneration theory. **C** M-(M + R) repair. **D** M-(M + U) and U-(U + M) repair

The circumflex nerve, musculocutaneous nerve, radial nerve (RN), ulnar nerve (UN), and median nerve (MN) are the five major targets in the repair of nerves for a severed upper extremity. Among these, the circumflex nerve [12] and musculocutaneous nerve [13] can both be functionally reconstructed using muscle transposition, free muscle transplantation or nerve transfer [14]. The three major nerves in the middle region of the upper limb are the MN, UN, and RN (Fig. 1A). If these nerves are defective, it is difficult to functionally correct them by the transposition of nerves or muscles remaining in the upper arm, which leads to severe wrist and hand dysfunction, significant restriction of fine movement, and even hand deformities [15, 16]. Therefore, there is an urgent need in the field of hand surgery for a method to repair multiple defects in nerves after avulsion amputation of the upper limb, especially defects in the RN, UN, and MN.

Functional recovery after end-to-side nerve repair is one of the manifestations of amplified nerve regeneration. In 1876, Despre inserted the distal end of a severed MN between the separated fibres of a UN and reported a certain degree of functional recovery after the operation [17, 18]. Studies have shown that the mechanism of end-to-side repair involves terminal and collateral sprouting [19–21]. In collateral sprouting, the regenerated axons grow along the side of the uninjured axons, while in terminal sprouting, axon regeneration occurs at the distal end of the injured or uninjured axons. Collateral sprouting can be facilitated by opening an epineurial window. Two types of magnified nerve regeneration via terminal sprouting have been reported in previous animal studies [22, 23]. In one type, the proximal end of a smaller nerve can innervate the distal ends of larger nerves. In the other type, the proximal end of one donor nerve trunk can be connected to the distal ends of two nerve trunks, leading to magnified nerve regeneration and subsequent innervation of the two nerve trunks. Zhang et al. reported the outcomes of the latter type of magnified nerve regeneration in an animal study in 2011 [22]. In rhesus monkeys, the proximal ends of the UN and musculocutaneous nerve were severed, and the proximal end of the UN was sutured with the distal ends of the UN and musculocutaneous nerve in a Y shape. After the operation, histopathological and neurophysiological examinations confirmed that the distal UN and musculocutaneous nerve had achieved a certain degree of reinnervation.

Based on the theory of magnified nerve regeneration (Fig. 1B), we proposed a clinical technique for repairing defects in multiple nerves after avulsion amputation of the upper limb. The nerve merging repair method relies on the use of fewer proximal nerves to innervate more distal nerves. First, a remaining (or abandoned) nerve is transplanted to the defective nerve. Next, the proximal end of one nerve and the distal ends of two nerves are sutured together in a Y-shaped connection; after the nerves are regenerated, the proximal nerve innervates two distal nerves. Thus, this method involves not only autogenous transplantation but also Y-shaped nerve coaptation and subsequent regeneration of nerves for the repair of RN, UN, and MN defects. The feasibility of the nerve merging repair method needs to be confirmed in clinical practice.

### Methods

This study was conducted in accordance with the Declaration of Helsinki. The ethics committee of Ningbo No. 6 Hospital approved this study. All the patients in this study were informed of the surgical plan and follow-up examinations in writing before surgery and signed an informed consent form.

## Nerve merging repair technique in upper limb replantation

The surgery for nerve merging repair can be divided into two scenarios according to whether the RN can undergo self-repair. The RN is the main nerve that controls extension of the wrist and fingers. Hence, a repair plan first needs to take into account whether RN self-repair is possible before the proximal and distal ends of nerves are directly sutured or subjected to coaptation.

When the RN suffers a horse-tail-like avulsion, it cannot undergo self-repair. A horse-tail-like avulsion means that the avulsion of the RN occurs from the trunk to the brachial plexus before the separation of the triceps brachii branches, and the triceps brachii branches are retained. These retained RN muscle branches are very important for elbow extension, and it is difficult to confirm the final level of the intact nerve trunk after a proximal avulsion injury. Therefore, we did not dissect the RN to a more proximal level to obtain an intact trunk to avoid damaging the residual triceps brachii branches. Another situation limiting RN self-repair is when the RN is avulsed at a very proximal level; in this case, finding the intact proximal end of the RN requires changing the patient’s position, which interferes with the replantation operation. When the RN cannot undergo self-repair, we propose a median-(median + radial) (M-(M + R)) repair approach by connecting the distal end of the RN to the proximal end of the MN (Table 1) (Fig. 1C), as described below. First, an appropriate residual segment of the UN is excised and transplanted to the distal defects of the RN and MN. Next, the proximal end of the MN and the distal ends of the MN and RN are sutured together in a Y shape. The suturing method for Y-shaped coaptation involves an epineurium-perineurium suture using 9 − 0 nylon thread, and the epineuriums on the adjacent surface of the two distal nerves are partially removed before suturing. Finally, the UN is set aside; because the UN has the worst expected outcome, the UN is sacrificed.

**Table 1:** Operating design for upper limb severing injury patients

Case	Point of severing	Initial trauma	Transplantation materials	Coaptation	Laying aside	Surgical plan	Principles of selection
Case 1	Upper arm	RN, MN, UN avulsed near brachial plexus, and RN cannot undergo self–repair	UN	MN, RN	UN	M–(M + R)	l horse–tail–like avulsion of RN, or RN avulsed at very proximal level l Balance between extensor and flexor l Sacrifice UN
Case 2	Elbow	RN, MN avulsed below the middle of the upper arm, UN avulsed more proximal	UN	MN, UN	–	M–(M + U)	l Select the thicker proximal nerve l Select the relatively intact proximal nerves l Repair all the distal nerves
Case 3	Upper arm		UN	MN, UN	–	M–(M + U)	
Case 4	Upper arm	RN, UN avulsed below the middle of the upper arm, MN avulsed more proximal	MN	UN, MN	–	U–(U + M)	
Case 5	Upper arm		MN	UN, MN	–	U–(U + M)	

**Fig. 2:**
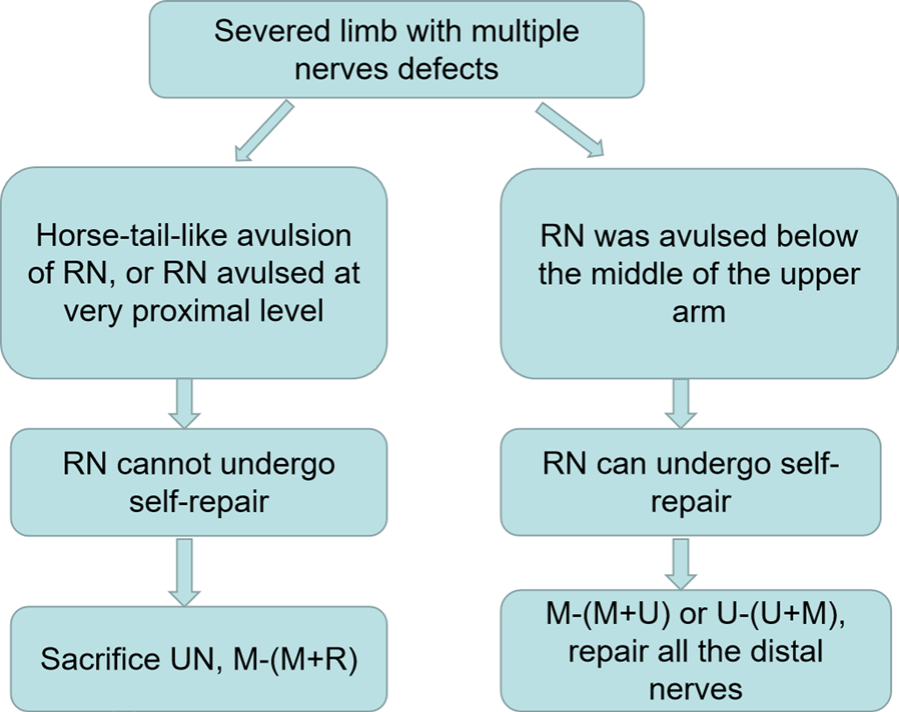
Flow chart showing the different types of proposed reconstruction

When the RN can undergo self-repair, there are two therapeutic options, i.e., median-(median + ulnar) (M-(M + U)) repair and ulnar-(ulnar + median) (U-(U + M)) repair (Table 1) (Fig. 1D). M-(M + U) repair is performed as follows: first, a residual portion of the UN of an appropriate length is excised and transplanted to the defect region of the RN to facilitate repair; next, the UN is trimmed and transplanted to the distal ends of the MN and UN; last, the proximal end of the MN is sutured to the distal ends of the MN and UN in a Y-shaped formation. U-(U + M) repair is performed in a similar manner. The two repair schemes were selected based on the results of intraoperative exploration using the following principles: (i) for proximal nerves, either the UN or MN is selected based on which is more intact, has only minor defects, or is thicker, and (ii) for distal nerves, the use of limited graft materials to repair all nerves is considered. A flow chart showing the different types of proposed reconstruction methods is shown in Fig. 2.

## Patients

Between April 2014 and September 2016, 5 patients with avulsion amputation of the elbow or upper arm with multiple nerve avulsion defects were treated at Ningbo No. 6 Hospital (Ningbo, China). The patients’ ages ranged from 31 to 56 years, with a mean age of 47.6 years. Four of the patients had avulsion amputation of the upper arm, and 1 had avulsion amputation of the elbow joint. The clinical information of the patients is shown in Table 1. The surgical plan included upper limb replantation combined with the nerve merging repair method.

## Follow-up and evaluation

Regular follow-ups were performed for all patients. The outcomes were investigated by assessing the following traits of the targets innervated by the distal nerves: muscle atrophy, skin sensation, and muscle strength. Additionally, we monitored for neuroma with pain at the end of the nerve and hand deformity. Finally, the Disabilities of the Arm, Shoulder, and Hand (DASH) scale [24] was used to evaluate the function of the ipsilateral upper limb. The muscles under the absolute innervation of the RN, MN, and UN are all forearm muscles, i.e., the extensor digitorum muscle, flexor carpi radialis muscle, and flexor carpi ulnaris muscle, respectively. The Medical Research Council (MRC) grading system [25] was used to assess the flexor and extensor muscle strength of the elbow, wrist, and fingers (thumb, index and middle finger). The skin areas under absolute innervation of the RN, MN, and UN are the first web space, thumb pulp, and little finger pulp, respectively [26].

## Results

All 5 patients with avulsion amputation of the elbow or upper arm and multiple nerve avulsion defects underwent replantation, and all replanted limbs survived. The mean follow-up duration was 67.0 months (51–80 months). The functional indicators for follow-up visits are summarized in Table 2. The range of sensory recovery in the distal absolute innervation area was S2-S3+. The degree of atrophy in the forearm muscles in the absolute innervation area was mild to moderate. The strength of the flexor and extensor muscles of the elbow was above M3, that of the flexor carpi muscle was between M3 and M4, and that of the extensor carpi muscle recovered to M2-M4. Some patients displayed relatively good recovery of the finger flexor and extensor muscle strength (to M4).

**Table 2:** Patients’ functional assessment during follow-up visits

	Case 1	Case 2	Case 3	Case 4	Case 5
Duration of follow–up (months)	76	72	80	51	56
Nerve/Sensory recovery in absolute innervation area	MN/S2	MN/S3+	MN/S3+	UN/S2	UN/S2
Nerve/Sensory recovery in absolute innervation area	RN/S2	UN/S2	UN/S3	MN/S2	MN/S3
Nerve/Atrophy of absolute innervating muscles	MN/mild	MN/mild	MN/mild	UN/moderate	UN/mild
Nerve/Atrophy of absolute innervating muscles	RN/moderate	UN/mild	UN/mild	MN/moderate	MN/moderate
Flexor muscle strength of elbow	M4	M4	M5	M4	M4
Extensor muscle strength of elbow	M4	M5	M5	M3	M4
Flexor muscle strength of carpi	M4	M4	M4	M3	M3
Extensor muscle strength of carpi	M2	M3	M4	M3	M4
Flexor muscle strength of fingers	M3	M1	M4	M3	M3
Extensor muscle strength of fingers	M1	M1	M4	M2	M3
Ape hand deformity	positive	negative	negative	positive	positive
Ulnar claw	positive	positive	positive	positive	negative
Neuroma with pain	No	No	No	No	No
DASH score	54	48	38	62	59

M0: no muscle contraction; M1: muscle fibrillation or contraction; M2: full-range motion, no gravity resistance; M3: capable of active motion of gravity resistance; M4: capable of active motion of gravity resistance and light obstruction resistance; M5: normal muscle strength. S0: sensory loss in the innervation region; S1: deep tactile sensation in the innervation area, recovery of pain sensation; S2: superficial tactile sensation in the innervation region, partial recovery of pain sensation; S3: recovery of tactile sensation and pain sensation in the innervation region, no hyperalgesia, S2PD > 15 mm, M2PD > 7 mm; S3+: recovery of sensation to S3 level and exhibiting a certain degree of two-point discrimination, S2PD: 7–15 mm, M2PD: 4–7 mm; S4: complete recovery of sensation, S2PD: 2–6 mm, M2PD: 2–3 mm

All 5 patients exhibited hand deformities (ape hand deformity and/or ulnar claw). The mean DASH score was 52.2 (38–62). All 5 patients experienced satisfactory recovery of skin sensation and elbow flexion and extension and partial recovery of hand muscle strength.

**Fig. 3:**
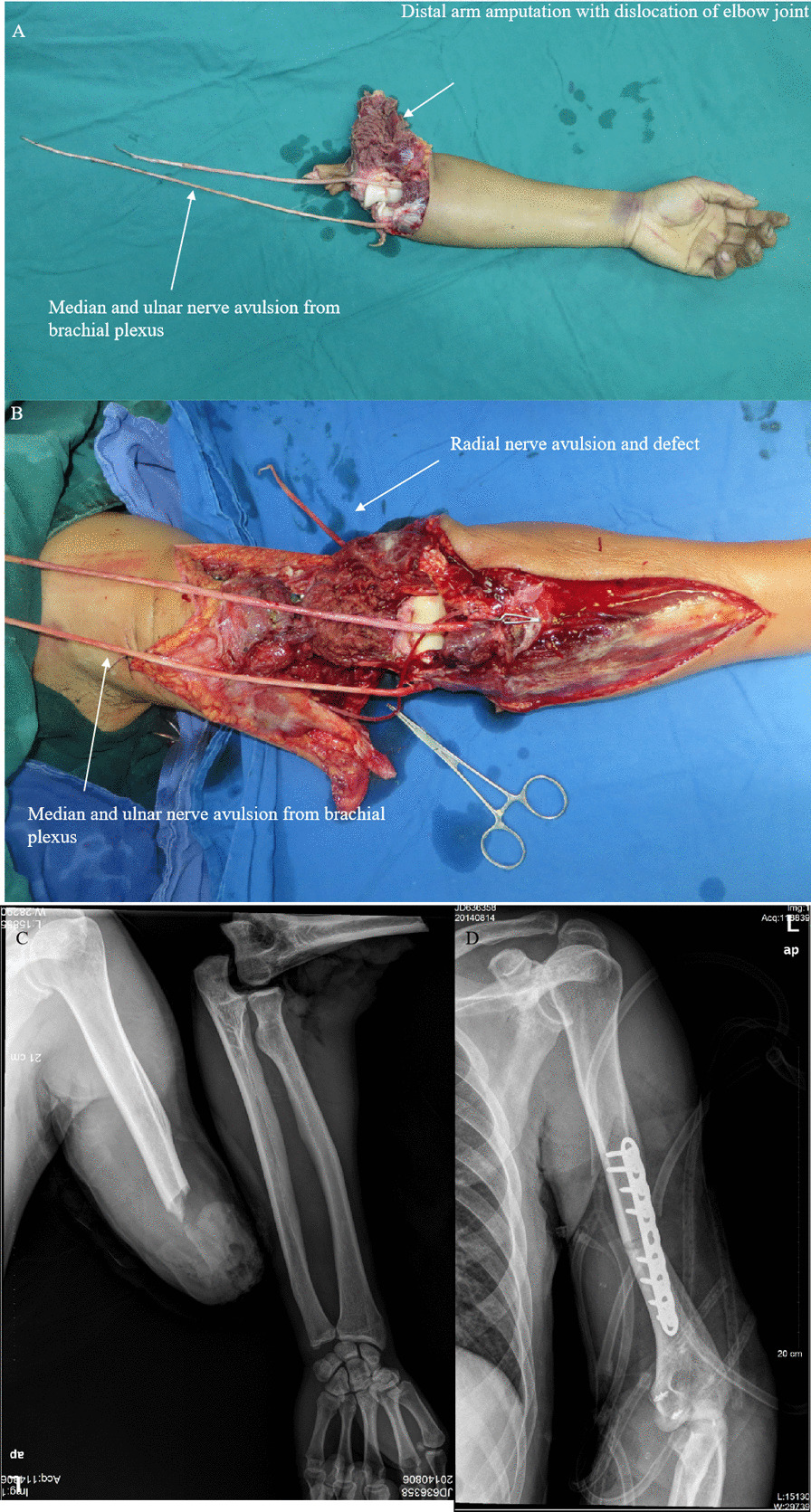
A patient (case 1) undergoing M-(M + R) repair (Part 1). **A** The left upper arm was severed, which was accompanied by dislocation of the elbow joint and avulsion of the three nerves with defects. **B** Temporary blood circulation was provided to shorten the warm ischaemia time, elbow joint reduction was performed, and the ligament was repaired. **C** Preoperative X-ray showed fractures in the distal humerus segment and dislocation of the elbow joint. **D** Postoperative X-ray showed internal fixation of the humeral fractures, reduction of the elbow joint, and implantation of anchor nails to repair the ligament

**Fig. 4:**
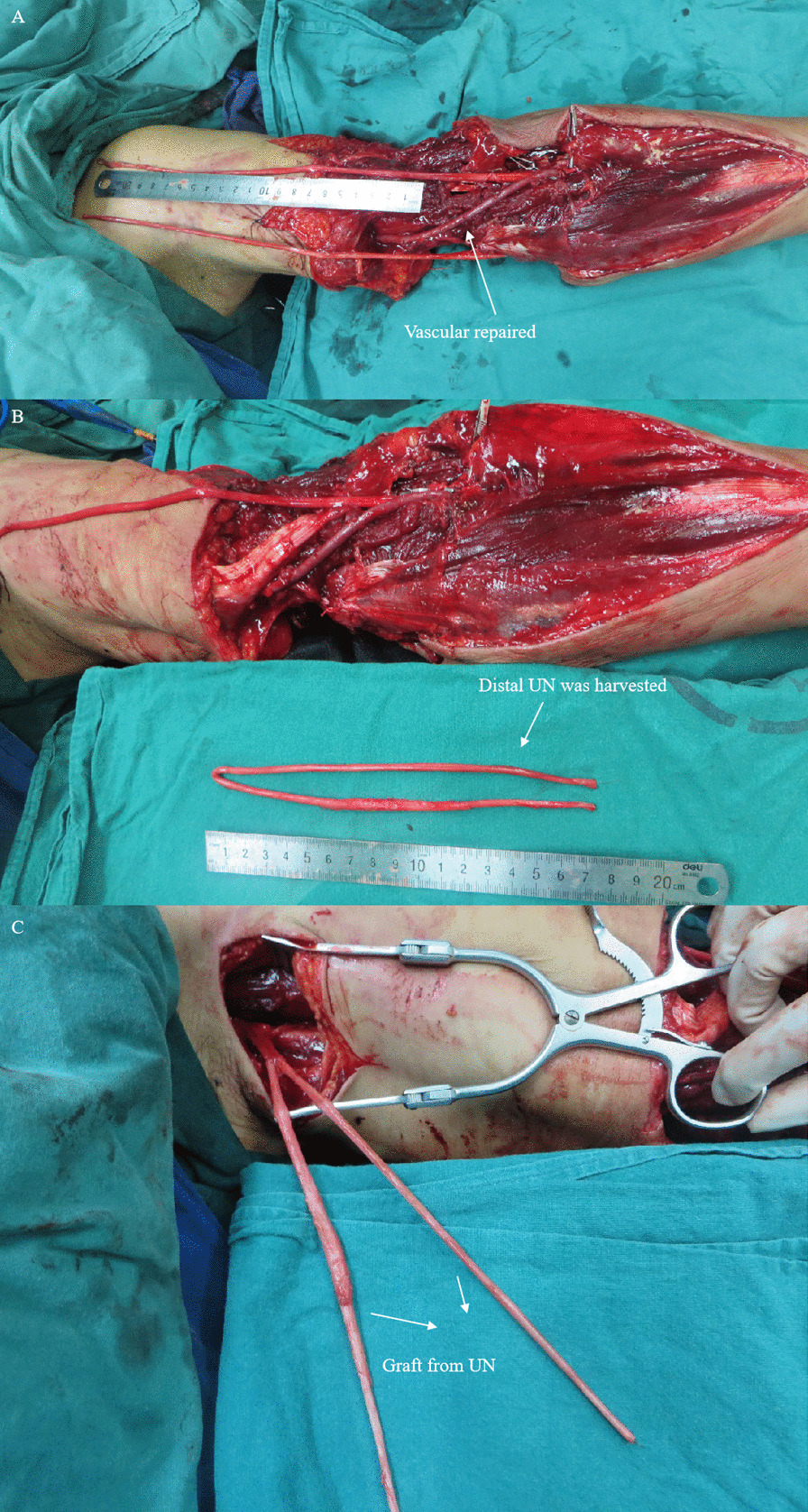
A patient (case 1) undergoing M-(M + R) repair (part 2). **A** Internal fixation of humeral fractures and repair of blood vessels. **B** The distal end of the UN was used as the graft material. **C** The graft material was cut into 2 segments, which were sutured with the starting point of the MN at the brachial plexus in a Y-shaped formation

**Fig. 5:**
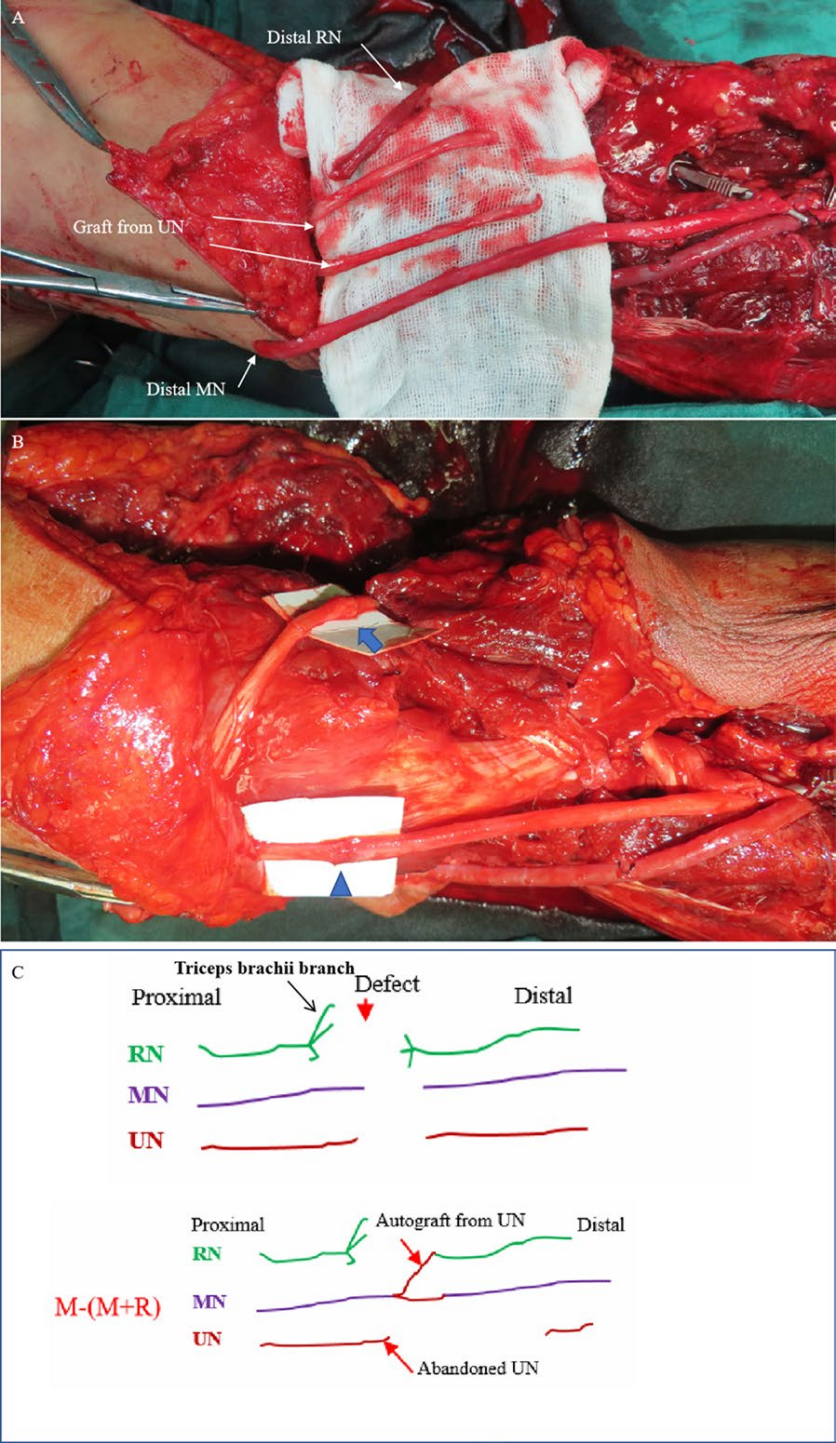
A patient (case 1) undergoing M-(M + R) repair (part 3). **A** The transplantation material was pulled through a subcutaneous tunnel to be sutured with the distal ends of the MN and RN. **B** The MN and RN were repaired by epineurium-perineurium suture. Arrow: RN. Arrowhead: MN. **C** Schematic diagram of nerve merging repair in case 1. The triceps brachii branch of the RN was intact; thus, the RN was not suitable for retrograde separation for self-repair

**Fig. 6:**
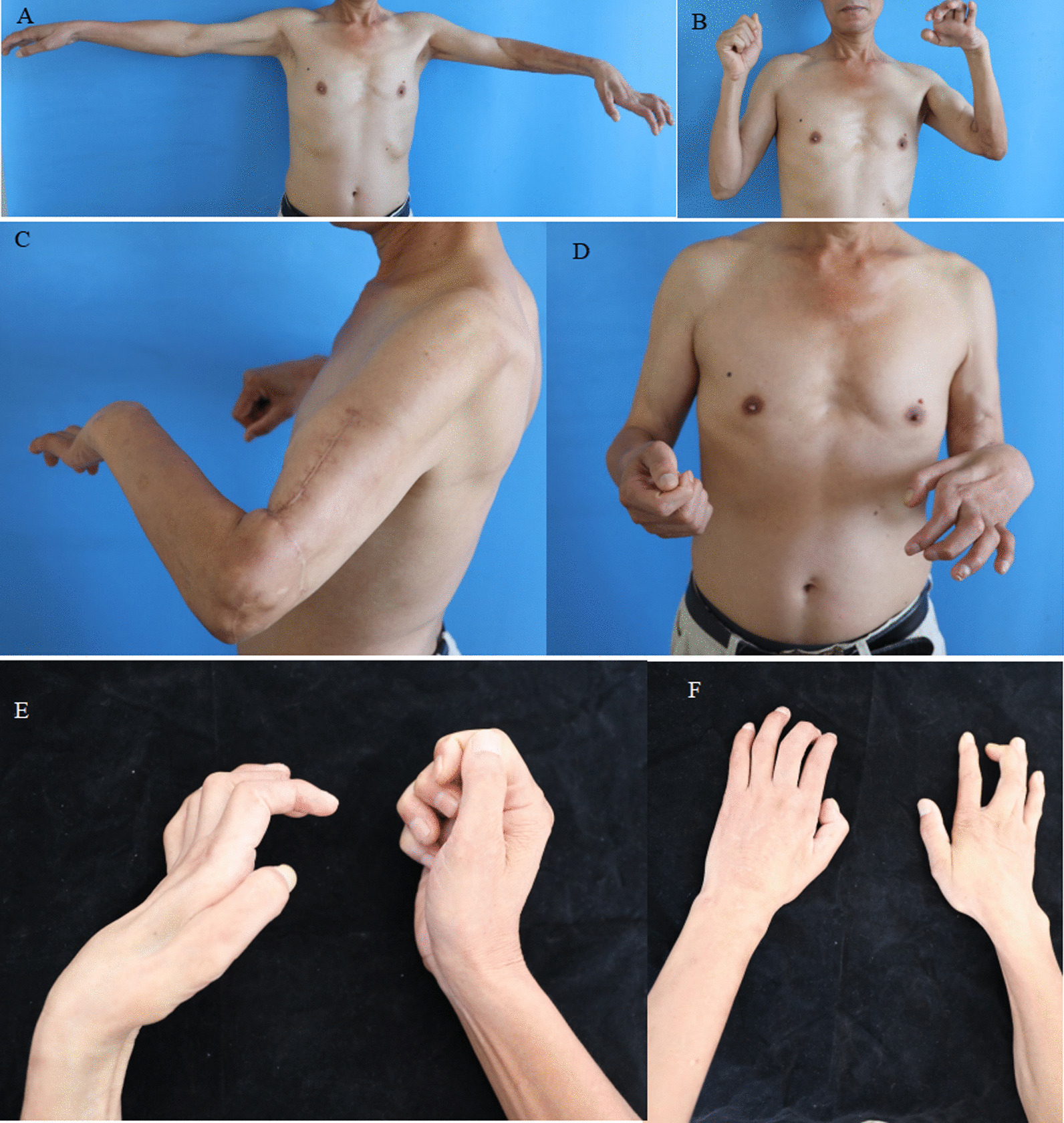
Follow-up examination for a patient who underwent M-(M + R) repair (case 1). **A**, **B** Good recovery of active flexion and extension in the left elbow joint. **C**, **D** The subject could complete left forearm pronation, but supination was limited; the intrinsic muscles of the left hand exhibited atrophy (due to laying the UN aside). **E**, **F** The subject could complete left wrist flexion, but wrist extension was limited; extension of fingers 1–5 was limited, and flexion of fingers was slightly limited

Figures 3, 4, 5 and 6 summarize the treatment process of a patient (case 1) undergoing M-(M + R) repair. For this patient, nerve merging repair and upper limb replantation were performed simultaneously. To maximize the repair outcome and reach a balance between the extensor carpi, finger extensors, flexor carpi, and finger flexors, the UN was designed to be sacrificed to repair the RN and MN. As such, M-(M + R) repair was employed for this patient. After nerve regeneration, the MN innervated the distal ends of the MN and RN.

**Fig. 7:**
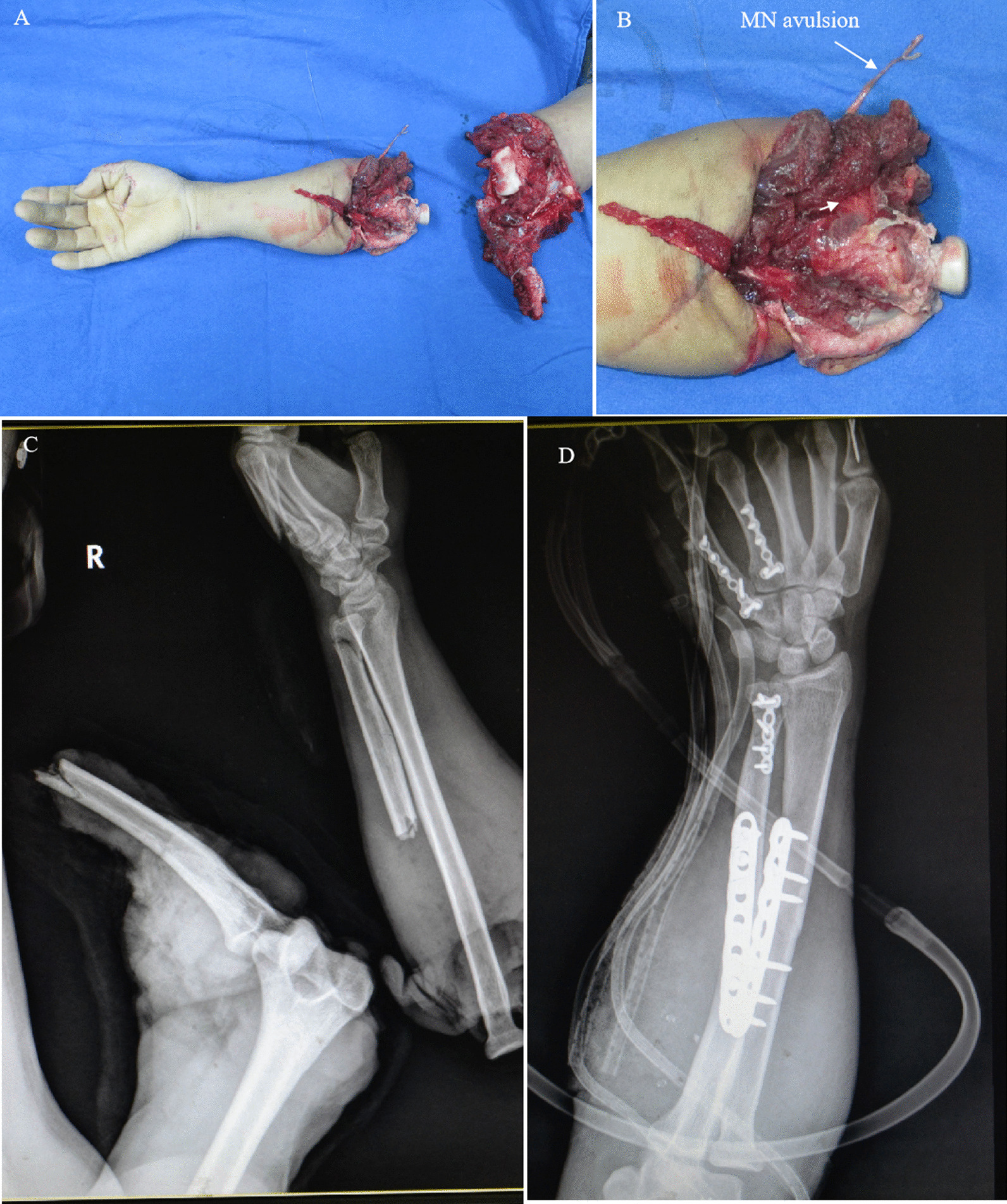
A patient (case 2) undergoing M-(M + U) repair (part 1). **A**, **B** The subject suffered avulsion amputation of the elbow joint and MN and UN in the right upper limb. **C** Preoperative X-ray showed that the subject had elbow joint dislocation and multiple fractures of the ulna and metacarpal bones. **D** Postoperative X-ray showed internal fixation of the ulna and metacarpal fractures, shortening and internal fixation of the radius, and reduction of the elbow joint

**Fig. 8:**
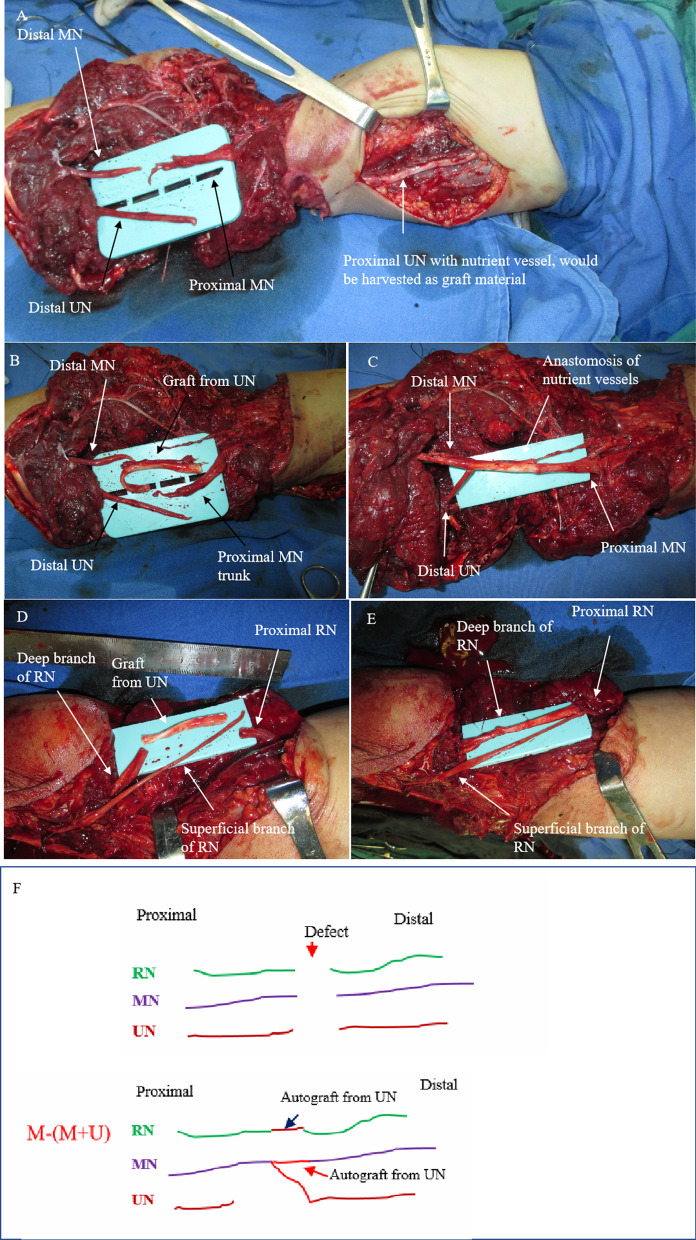
A patient (case 2) undergoing M-(M + U) repair (part 2). **A **The fracture was fixed; there were long-segment defects in the MN and UN and deep branch of the RN. **B** The proximal trunk of the UN containing nutrient vessels was used as the graft material. **C** The proximal end of the MN was sutured to the distal ends of the MN and UN, and the nutrient vessel grafts were anastomosed. **D** The UN trunk was used as the graft material to repair a deep RN branch. **E** The superficial RN branch was repaired by direct suturing. **F** Schematic diagram of nerve merging repair in case 2

**Fig. 9:**
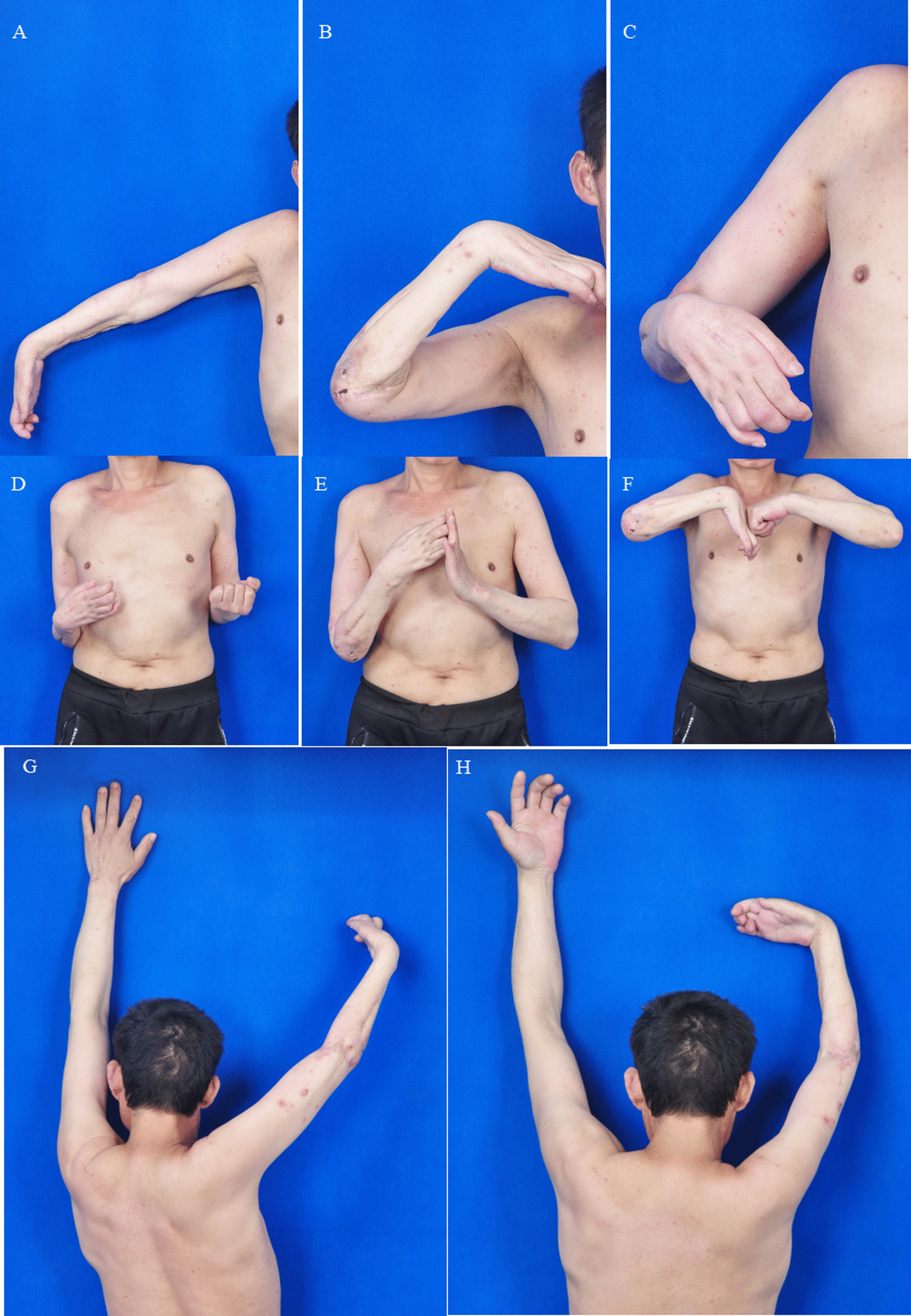
Follow-up examination of a patient who underwent M-(M + U) repair (case 2). **A**, **B** The subject displayed overall normal performance in active flexion and extension of the right elbow joint. **C**, **D** Right forearm pronation and supination were partially limited. **E**–**H** The right wrist joint displayed normal active flexion but partially restricted extension. The active motion of fingers 1–5 was limited

Figures 7, 8 and 9 show the treatment process of a patient (case 2) undergoing M-(M + U) repair. The patient showed the following signs: deep RN branch defects, superficial RN branch ruptures, avulsion and long-segment MN and UN loss, and a relatively intact proximal MN end. Based on these findings, M-(M + U) repair was proposed. Autogenous nerve transplantation was first performed for the RN before coaptation was conducted to facilitate innervation of the distal ends of the MN and UN by the MN.

## Discussion

At present, the main methods for treating long-segment defects of a single nerve are autologous nerve grafting, allogeneic nerve grafting [27], and artificial nerve conduit bridging [28], among other methods [29]. Although different therapeutic effects have been reported, there have been few studies on the repair of nerve defects longer than 6 cm [10]. Avulsion amputation of the upper limb is accompanied by multiple nerve avulsions and defects. However, it is not realistic to repair every nerve via transplantation repair for three reasons: the limited material available in the autogenous nerve graft donor area; the infeasibility of vascularized nerve transplantation due to an insufficient number of blood vessels in the recipient area for anastomosis; and the poor outcomes of long-segment allogeneic nerve transplantation. Based on the theory of magnified nerve regeneration, a new approach called nerve merging repair is proposed to overcome the challenges of surgically treating defects in multiple nerves. If the RN cannot undergo self-repair (via direct suturing or bridging), M-(M + R) repair is employed; alternatively, if the RN can undergo self-repair, M-(M + U) or U-(U + M) is used. The 5 patients treated in this study all underwent nerve merging repair, which led to sensory function improvement and partial motor function recovery in the affected limb. As such, we developed a surgical approach that solves the problem of repairing long-segment defects spanning multiple nerves after avulsion of the upper limb. The significance of the study includes the following: (i) the approach clinically follows the theory of magnified nerve regeneration; (ii) the approach not only allows alleviation of target muscle atrophy and considerable upper limb function recovery but also causes no additional functional loss in the nerve donor area.

The phenomenon of reinnervation we observed in this study is consistent with the theory of magnified nerve regeneration. The follow-up results (Table 2) showed that the range of sensory recovery in the distal absolute innervation area was S2-S3+, that the degree of atrophy of the forearm muscle in the absolute innervation area was mild to moderate, and that the recovered strength of some target muscles was M4. All of the coapted distal nerves were innervated, consistent with the theory of magnified nerve regeneration. Although high-level reinnervation cannot easily be achieved by this method, its outcome is superior to that of laying nerves aside. Although the level of motor and sensory function recovery was low, the patients were able to perform some of their daily life and work activities. Finally, no cases of neuroma with pain in a proximal nerve occurred in any of the 5 patients.

Other advantages of the nerve merging repair method reported herein are as follows: (i) compared with secondary nerve repair, this method drastically shortens the denervation time of the target muscles; and (ii) when the survival of the replanted tissue is not clear, this method can be applied to repair the main nerves of the upper limb and avoid causing damage to the donor area by autologous sural nerve grafting.

Finally, our method can effectively restore partial upper limb function. After replantation, even though the functions of the affected limbs had not recovered to ideal levels, some of the muscles had regained innervation, which provided more options for later functional reconstruction (tendon transposition) to achieve improved abilities. For example, in case 1, because the patient recovered to M4 flexor muscle strength and M2 extensor muscle strength, two options to further improve hand function could be implemented in later stages: (i) the wrist joint could be fused in a functional position, and flexor carpi tendon transfer could be performed to reconstruct finger extension function [30, 31], and (ii) alternatively, free functional muscle transfer could be performed to re-establish the ability for wrist and finger extension (motor branches of the free muscle could be sutured selectively to motor branches of the flexor carpi muscle to restore nerve innervation) [13, 32].

The limitations of this study are as follows: (1) as the sample size of this study is small and this was not a case-control study, the evidence is insufficient to prove the theory of amplified nerve regeneration, and (2) despite this intervention, the level of neurological function recovery after limb replantation remained low. In the future, the combined use of medicine and technique [33, 34] to promote nerve regeneration may help to improve function.

## Conclusions

To a certain extent, treatment with the nerve merging repair method improved the sensory and motor function of the affected limb and limited the loss of function of the donor nerve area. This intervention could serve as a new approach for repairing long-segment defects in multiple nerves caused by avulsion amputation of the upper limb.

## Data Availability

All data generated or analysed during this study are included in this published article.
